# Parasites in the City: Degree of Urbanization Predicts Poxvirus and Coccidian Infections in House Finches (*Haemorhous mexicanus*)

**DOI:** 10.1371/journal.pone.0086747

**Published:** 2014-02-04

**Authors:** Mathieu Giraudeau, Melanie Mousel, Stevan Earl, Kevin McGraw

**Affiliations:** 1 School of Life Sciences, Arizona State University, Tempe, Arizona, United States of America; 2 Global Institute of Sustainability, Arizona State University, Tempe, Arizona, United States of America; University of Georgia, United States of America

## Abstract

**Background:**

Urbanization can strongly impact the physiology, behavior, and fitness of animals. Conditions in cities may also promote the transmission and success of animal parasites and pathogens. However, to date, no studies have examined variation in the prevalence or severity of several distinct pathogens/parasites along a gradient of urbanization in animals or if these infections increase physiological stress in urban populations.

**Methodology/Principal Findings:**

Here, we measured the prevalence and severity of infection with intestinal coccidians (*Isospora* sp.) and the canarypox virus (*Avipoxvirus*) along an urban-to-rural gradient in wild male house finches (*Haemorhous mexicanus*). In addition, we quantified an important stress indicator in animals (oxidative stress) and several axes of urbanization, including human population density and land-use patterns within a 1 km radius of each trapping site. Prevalence of poxvirus infection and severity of coccidial infection were significantly associated with the degree of urbanization, with an increase of infection in more urban areas. The degrees of infection by the two parasites were not correlated along the urban-rural gradient. Finally, levels of oxidative damage in plasma were not associated with infection or with urbanization metrics.

**Conclusion/Significance:**

These results indicate that the physical presence of humans in cities and the associated altered urban landscape characteristics are associated with increased infections with both a virus and a gastrointestinal parasite in this common songbird resident of North American cities. Though we failed to find elevations in urban- or parasite/pathogen-mediated oxidative stress, humans may facilitate infections in these birds via bird feeders (i.e. horizontal disease transmission due to unsanitary surfaces and/or elevations in host population densities) and/or via elevations in other forms of physiological stress (e.g. corticosterone, nutritional).

## Introduction

More than half of the world’s population now lives in cities [1], and rapid urbanization in the last few hundred years has dramatically altered natural habitat structure, ecosystem functioning, and faunal and floral biodiversity [2] [3] [4]. Most studies of urban impacts on wildlife historically have centered on censusing populations and monitoring trends in species distributions and richness [3]. However, there has been a recent scientific push to understand finer-scale effects of cities on the ecology, physiology, health, and behavior of individual animals [4] [5] [6]. Information on how, for example, stress, disease, expression of ornamental traits, and behavioral plasticity are linked to urbanization can give key insights into short-term urban impacts on the fitness of organisms (which have obvious long-term ties to population persistence).

Current interest in urban wildlife diseases is far-reaching, as it has direct implications for the spread of zoonoses in humans (which now account for 75% of the world’s emerging infectious diseases) [7] and domestic animals. Due to their broad life-history effects on development, survival, and reproduction [8] [9], parasites and pathogens have also been argued to contribute to the success of plant and animals species in colonizing or persisting in urban areas [10] [11]. Population thinning in cities can decrease opportunities for horizontal pathogen/parasite transmission, though some animals that thrive in cities may experience the opposite [12]. Also, disease severity may rise in city-dwelling animals due to the physiological stresses (e.g. pollution, impaired nutrition, glucocorticoid elevation, compromised immune system) of urban life [13] [4] [14].

To date, comparisons of disease between urban and rural animal populations have yielded mixed results. Some studies have shown reduced parasitism in urban animals (e.g. blackbirds, *Turdus merula*; [15] [16] [17] [18] [19]; see [5] for a multi-species avian study) compared to their rural counterparts, while others have found higher pathogen/parasite abundance in cities (e.g. woodchucks (*Marmota monax*) [20]; bumblebees (*Bombus terrestris*) [21]; mice (*Peromyscus spp*) [22]; northern cardinal (*Cardinalis cardinalis*) [23]). However, most of these prior studies center in on only one type of parasite or pathogen and do not consider the severity of their effects on hosts. Moreover, they fail to integrate detailed information on the type or degree of urbanization (e.g. land use types, human population demographics), most often simply categorizing habitats for comparison as “urban” or “rural”. Gathering additional information on the host, parasites, and environmental characteristics is central to developing an integrated framework for understanding if and how different animals in urban environments should be influenced by disease.

To the best of our knowledge, no studies to date have examined infection prevalence or severity by multiple parasites/pathogens along a gradient of urbanization. Free-living animals often carry several parasites species [24] and these multiple infections may increase or decrease parasite virulence [25] [26] [27] [28]. For example, a decrease of parasite virulence may occur when an infection with a first parasite provide cross-immunity against other parasites [29] [30]. However, multiple infections have been shown to often cause detrimental effects such as anaemia, reduced body mass and survival in many host–parasite systems [31] [32].

In addition, we know of no work linking parasites, urbanization, and stress. Among the many means of assessing stress in animals, oxidative stress has emerged in recent years as an under-studied component of the stress response system of wild animals [33], including in relation to urbanization [34]. Urban pollution elevates oxidative stress in humans [35] [36] and birds [34]. Nutritional shifts in urban environments may lead to changes in dietary antioxidant availability and thus the capacity to counteract free-radical damage. For example, Isaksson and Andersson [37] showed that dietary carotenoid concentration was significantly lower in the urban caterpillars that constitute an important component of the diet of great tits (*Parus major*). Finally, it is possible that elevated parasitism (and/or associated immune system activation) may increase oxidative stress in urban animals [38].

In order to assess how the rapid changes of land use in urban areas are associated with modifications of the host-pathogen interaction, we assessed the degree of infection by the avian poxvirus (canarypox, genus *Avipoxvirus*) [39] and by coccidian parasites (*Isospora* sp.) [40] in male house finches (*Haemorhous mexicanus*) at seven sites along a gradient of urbanization in the city of Phoenix, Arizona, USA. House finches are native residents of the desert southwestern United States but are now abundant inhabitants of cities around North America [41]. Poxvirus is a common avian virus and manifests in lesions typically restricted to the feet, eye, nares, wing, and ears and in the late stages become bloody and crusty and may lead to loss of digits [42]. Coccidians inhabit the gut lining of finches [40] and, as in other birds, are thought to disrupt nutrient uptake and result in shedding oocysts via feces [43] [44] [45] [46] [47]. Both pox and coccidia are known to have strong negative effects on expression of a key fitness trait (sexually selected plumage coloration) in house finches [48] [40]. We also quantified thirteen different metrics of urbanization, including human population density and twelve measurements describing land-use patterns within the 1 km radius around each of our trapping sites, to examine if or how these factors associate with pox or coccidia infections. Finally, we assessed the potential relationship between oxidative stress, the degree of urbanization, and parasite infections in order to determine how a potential increase of parasite infection in urban areas is associated with increased stress levels.

## Methods

### Ethics Statement

All experiments were carried out under United States Fish and Wildlife Service permit #MB088806-1 and Arizona State Game and Fish scientific collecting permit SP727468. All experimental procedures were approved by the Institutional Animal Care and Use Committee at Arizona State University (Protocol # 12–1234).

### Field Methods

From 16 August–15 September 2011, we used basket traps and Potter traps baited with sunflower seeds [49] to capture 174 first-year male house finches from 7 sites along a gradient of urbanization in the Phoenix metropolitan area. These sites varied from 1–35 km to the urban Phoenix center and in many other environmental aspects (Table 1). We obtained permits from the Phoenix zoo, the Arizona State University, the city of Gilbert, the rangers of the Estrella and South Mountain Regional Parks to trap finches in their parks. For the Phoenix downtown and Mesa sites, we trapped in a private backyard where the owner of the land provided permission to conduct the study. The field studies did not involve endangered or protected species.

**Table 1:** Characteristics of the sites at which we studied house finches in Maricopa County, USA.

Capture site	Phoenixdowntown	ASU campus	Mesa OrganicFarm	CrossroadsDistrict Park	Phoenix Zoo	South MountainPark	Estrella MountainPark
**City**	**Phoenix**	**Tempe**	**Mesa**	**Gilbert**	**Phoenix**	**Tempe**	**Goodyear**
**Geographical coordinates**	33° 27′ N 112° 03′ W	33° 25′ N111° 55′ W	33° 27′ N111° 49′ W	33° 19′ N111° 43′ W	33° 27 ’N 111° 57′ W	33° 21′ N112° 4′ W	33° 25′ N112° 25′ W
**Human population living within** **1** **km of study site**	7291	10385	4600	17175	50	1001	11
**Sample size** *	24 (18, 19, 20)	36 (22, 36, 20)	21 (20, 21, 18)	29 (20, 27, 22)	21 (18, 21, 21)	22 (20, 22, 22)	20 (20, 20, 20)
**Habitats (% of land covered):**							
**Cultivated Vegetation**	0.00	0.00	3.44	7.62	0.00	0.00	0.00
**Cultivated Grass**	0.00	0.00	0.00	1.45	8.05	0.05	1.11
**River Gravel**	0.00	0.00	0.00	1.53	1.14	0.00	19.00
**Vegetation**	1.01	2.25	6.37	3.32	3.16	4.12	3.62
**Disturbed (Commercial/Industrial)**	8.94	23.63	13.91	11.72	5.02	2.80	1.16
**Disturbed (Asphalt)**	21.17	24.75	8.60	9.59	13.62	5.05	1.71
**Undisturbed**	10.55	9.56	14.45	12.05	57.45	68.89	67.44
**Disturbed (Compacted Soil** **prior Agriculture)**	0.00	0.00	3.00	9.56	0.00	0.00	0.00
**Disturbed (Compacted Soil)**	0.78	1.01	1.50	2.44	1.99	1.81	0.67
**Disturbed (Mesic Residential)**	8.03	18.84	23.93	13.66	3.47	4.64	0.36
**Disturbed (Xeric Residential)**	49.52	19.95	24.79	26.62	5.54	12.62	1.37
**Water**	0.00	0.00	0.00	0.44	0.54	0.03	3.55

* Number of birds trapped and studied for our measurements of coccidian parasites, avian pox, and oxidative damage respectively.

At capture, each bird was leg banded with a numbered United States Geological Survey metal tag for individual identification. We also collected 150 µl of whole blood through the alar vein with heparinized capillary tubes. Blood was centrifuged (10000 g for 3 min) and the plasma saved at −80°C for later analysis of oxidative stress. Finally, we determined body mass (to the nearest 0.1 g with a digital scale) and tarsus length (to the nearest 0.1 mm with digital calipers) so we could calculate body condition (using the residuals of the mass/tarsus linear regression: F_1,187_ = 31.75, P<10^−6^) for comparison to parasite burden, oxidative stress, and urbanization metrics. After capture, birds were temporarily housed individually in small cages (26.7×21.6×34.3 cm) with *ad libitum* access to food and water at our animal facility until fecal collection that afternoon (see more below), at which point birds were returned to their trapping sites and released.

### Quantifying Poxvirus and Coccidia

Infection by canarypox virus [39] was visually scored at capture on a scale of 0 to 4, adapted from Thompson *et al*. [48]: 0 =  no infection (no lesions); 1 =  minor infection, with one small lesion on the head or foot; 2 =  moderate infection, with a few small lesions on the head or feet; 3 =  major infection, with one large lesion on the head or feet; and 4 =  extreme infection, with several large lesions on the head and feet.

We monitored coccidian abundance via standard fecal collection and float methods [50]. Starting at 1600 hrs. on the day of capture, each bird was kept in a small paper bag for up to 1.5 hours, after which point feces were collected from the bottom of the bag and stored in 2.2% potassium dichromate until laboratory analyses. Fecal float and microscope-slide preparations follow those used by Brawner *et al.* [40] and McGraw and Hill [50]. Parasite load was estimated on an integer scale from 0–5∶0 =  no oocysts present; 1 = 1–10 oocysts present; 2 = 11–100 oocysts present; 3 = 101–1000 oocysts present; 4 = 1001–10000 oocysts present; and 5 =  greater than 10000 oocysts present.

We calculated the prevalence (number of birds infected/number of birds examined) and severity (mean infection score for infected birds only) of coccidian infection at each study site. We only calculated prevalence for poxvirus infections because the number of birds infected at each site was too low to calculate severity.

### Measurement of Oxidative Stress

Oxidative damage was measured in plasma using a miniaturized thiobarbituric acid reactive substances (TBARS) assay modified from a commercially available kit (Oxi-Tek TBARS assay kit, ZeptoMetrix Corp., Buffalo, NY). This commonly used TBARS method quantifies oxidative stress by measuring levels of lipid peroxidation, a major biomarker of oxidative stress in animal tissues [51] [52] [53] [54]. Specifically, this assay involves the reaction of malondialdehyde (MDA), a naturally occurring product of lipid peroxidation, with thiobarbituric acid (TBA) under conditions of high temperature and acidity to generate an adduct that can be measured by spectrophotometry. Briefly, 20 µL thawed plasma was mixed with 20 µL 8.1% sodium dodecyl sulfate (SDS) and 500 µL TBA buffer reagent. The TBA buffer reagent was prepared by mixing 50 mg TBA with 10 ml acetic acid and 10 ml NaOH. Samples were then vortexed and incubated at 95°C in capped tubes for 60 minutes. Thereafter, the sample was placed on ice for 10 minutes and centrifuged at 3000 rpm for 15 minutes. After centrifugation, the supernatant was removed and absorbance measured at 532 nm (Bio-Tek µQuant microplate spectrophotometer, Winooski, Vermont, USA). Sample concentrations were calculated by interpolation from a standard curve of MDA in concentration from 0–100 nmol·ml^−1^ and are expressed in nmol·ml^−1^ of MDA equivalents. Higher values correspond to greater oxidative damage.

### Metrics of Urbanization

To develop a broad and ecologically relevant framework for examining links between disease, oxidative stress, and specific attributes of urbanization, we obtained several urban parameters from a local database that is part of the Central-Arizona-Phoenix Long Term Ecological Research program [55] [56] [57] : (1) human population density within a 1 km radius around each trapping site, estimated from the 2010 US Census data; (2) extent of land use and land cover (LULC) within a 1 km radius around each trapping site. We extracted the 12 following LULC types: % cultivated vegetation, cultivated grass, river gravel, vegetation, disturbed-commercial/industrial, disturbed-asphalt, undisturbed, disturbed-compacted soil prior agriculture, disturbed-compacted soil, disturbed-mesic vegetation residential, disturbed-xeric vegetation residential, and water (see [55] [56] [57] for a full description of the LULC types). Using principal component analysis (PCA), we collapsed these 13 urbanization scores into three PCs that summarized >80% of urban environmental variation (PC1 = 44%, PC2 = 23%, PC3 = 14%). PCA1 loaded positively and strongly (component loading >85%) on the percentage of land covered by undisturbed habitat and on human population density. PCA2 loaded positively and strongly (component loading >76%) on the percentage of land covered by cultivated vegetation and on the percentage of land covered by compacted soil (prior agriculture or not). Finally, PCA3 loaded positively on the percentage of land covered by cultivated grass (component loading >71%).

### Statistics

All statistical analyses were carried out using Statistica software (StatSoft, Inc. Tulsa, USA) with α set at 0.05. We ran non-parametric Spearman correlations between the urbanization metrics extracted from the PCA and the average values for parasite infections (coccidia severity and prevalence, poxvirus prevalence), tarsus length, body mass, oxidative stress and body condition of each population.

## Results

Mean body condition and tarsus length at each site did not associate with our metrics of urbanization (all P>0.15). However, body mass was significantly positively associated with urbanization PCA2 scores (rho = 0.82, P = 0.02) but not with other PCs (PC1: rho = 0.14, P = 0.76; PC3: rho = −0.64, P = 0.12). Thus, birds were heavier at sites with more land covered by compacted soil and cultivated vegetation.

### Coccidian Infection

Severity and prevalence of infection by coccidians tended to be positively correlated within our study sites (rho = 0.75, n = 7, P = 0.0.052). In addition, infection prevalence by the two parasites was not correlated along our gradient of urbanization (rho = 0.38, P = 0.40).

**Figure 1:**
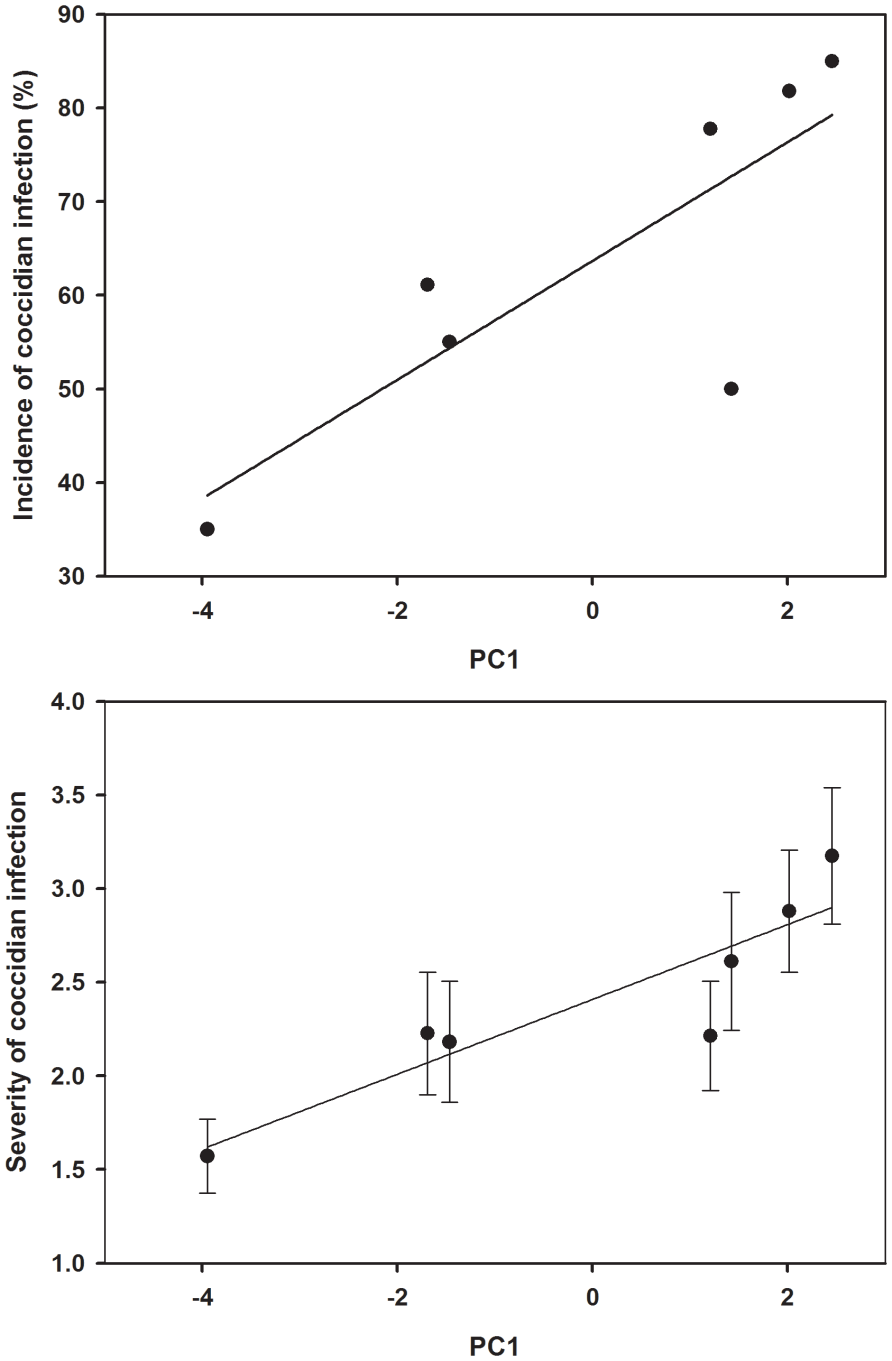
Relationships between an urbanization metric (PC1) measured within a 1-km radius around each trapping site and the (A) prevalence and (B) severity of infection by coccidian parasites (±SE) in male house finches. Prevalence and severity of infection increase in more human populated areas with less land covered by natural habitat.

Prevalence of coccidian infection tended to be associated positively with urbanization PC1 score (rho = 0.75, P = 0.052; Figure 1) but was not linked to PC2 (rho = −0.11, P = 0.82) or PC3 (rho = 0.32, P = 0.48). Severity of coccidian infection was significantly associated with urbanization PC1 score (rho = 0.89, P = 0.006; Figure 1) but not with PC2 (rho = 0.28, P = 0.53) or PC3 (rho = −0.03, P = 0.94). Taken together, these results indicate that coccidian prevalence and severity tended to be higher in more human-populated areas with more disturbed habitat. Finally, at the individual level, the severity of infection by coccidians was significantly and positively related with body mass (rho = 0.16, P = 0.04); heavier birds were more infected by this parasite.

### Poxvirus Infection

**Figure 2:**
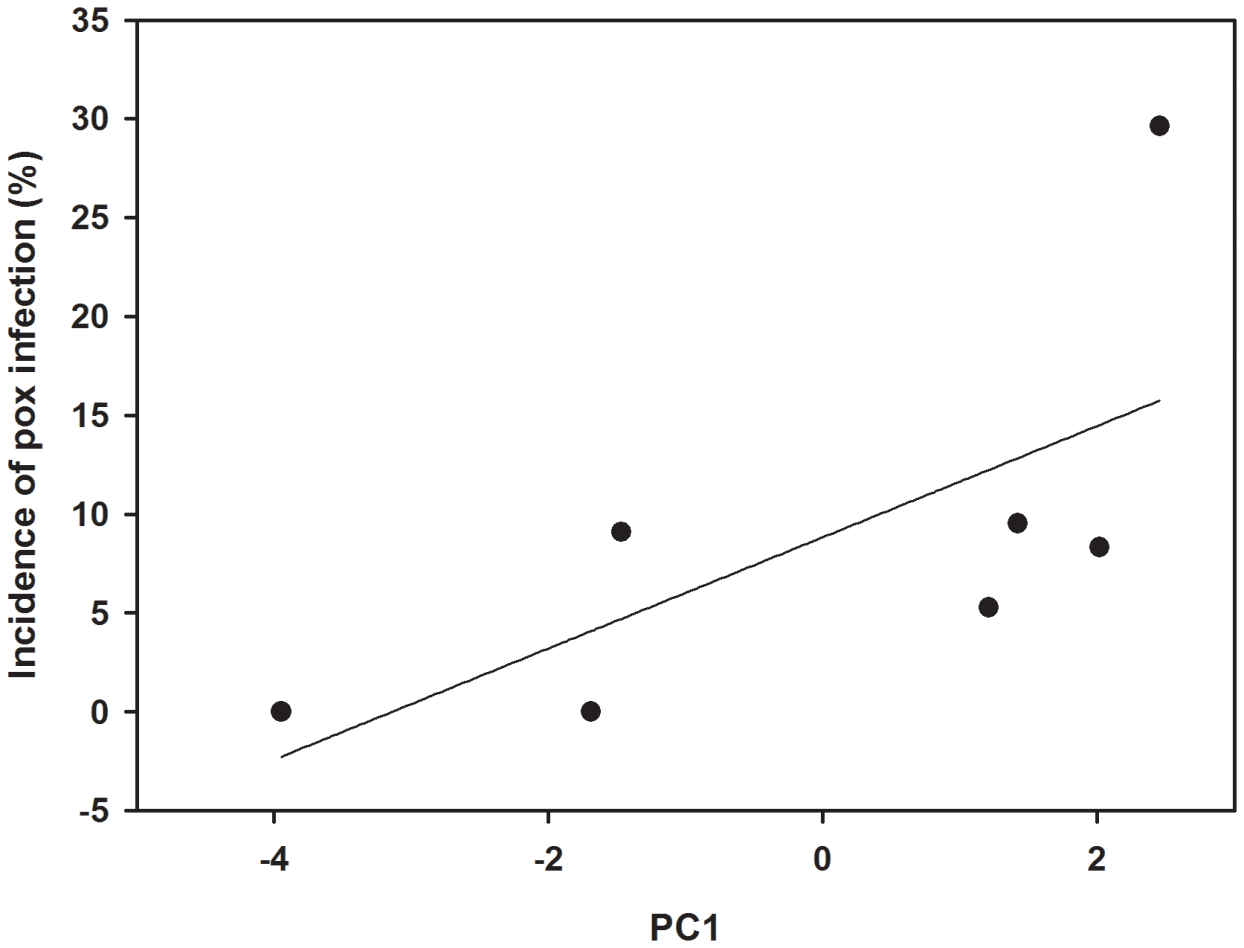
Relationship between an urbanization metric (PC1) measured within the 1-km radius around each trapping site and the prevalence with which house finches were infected by the canary poxvirus.

Prevalence of poxvirus was significantly positively associated with urbanization PC1 scores (rho = 0.81, P = 0.03; Figure 2) and tended to associate with PC2 scores (rho = 0.70, P = 0.08) but was not linked with PC3 (rho = −0.21, P = 0.64). Thus, the percentage of birds infected with poxvirus was also higher in areas more populated by humans and with more disturbed habitat. At the individual level, the severity of infection by the poxvirus was not related to body mass (rho = 0.01, P = 0.85).

### Oxidative Stress

Mean level of oxidative stress at each site was not associated with any of our urbanization (PC1: rho = −0.21, P = 0.64; PC2: rho = −0.43, P = 0.34; PC3: rho = −0.075, P = 0.06) or infection metrics (coccidia prevalence: rho = –0.50, P = 0.25; coccidian severity: rho = −0.21, P = 0.64; pox prevalence: rho = −0.01, P = 0.99). At the individual level, the levels of oxidative stress were not associated with the degree of infection by coccidian (rho = −0.06, P = 0.47), by the poxvirus (rho = −0.002, P = 0.98) or body mass (rho = 0.04, P = 0.59).

## Discussion

Here we show for the first time in animals that the degree of infection by two distinct parasites is associated with an increase of anthropogenic activities. Specifically, we found that the severity of coccidian infection and the prevalence of poxvirus infection increased in house finches in relation to human population density and was inversely related to the proportion of undisturbed habitat in the Phoenix metropolitan area. None of our other metrics of urbanization were associated with the degree of infection by the two parasites measured, suggesting that a decrease in natural land cover associated with human development is the driving force behind the increase in urban parasitism.

Our results contrast with many prior studies that have found a lower degree of infection in urban birds [15] [16] [17] [19] [18] [5]. However, several recent studies that have considered gastrointestinal parasites, such as the coccidians that we studied here, showed that endoparasite infections in mice, bumblebees, and Cooper’s hawks increase in urban environments ([58] [22] [21] but see [59] [18] for opposite pattern with helminth parasites in red fox and blackbirds). Thus, it is possible that urban disease patterns depend upon the types of parasites involved and their transmission methods. In addition, intestinal parasitism may increase more in urban areas because higher densities of animals in these areas (e.g. around specific sources of food, like feeders or waste) may elevate the transmission of these parasites by direct contact, an oral-fecal route, or by increasing the proportion of intermediate hosts infected [12]. In accordance with this hypothesis, monthly estimates of house finch flock sizes showed that winter flock sizes are associated positively with average monthly prevalence of the bacterial disease *Mycoplasma gallisepticum* [60]. In addition, house finches had a greater tendency to flock with an increase in density in more urbanized habitats [61], suggesting that the higher concentrations of birds in specific places (e.g. feeders, parks) in urban areas are an important driver of parasite transmission in the city. It is also noteworthy that the other disease we studied here, avian poxvirus, can also be transmitted horizontally (though also from biting insect vectors) [62]. Consistent with our hypothesis, an experimental clumping of resources resulted in elevated densities of wild raccoons (*Procyon lotor*) and increased prevalence of their gastro-intestinal parasite roundworm *Baylisascaris procyonis* [63].

In addition to disease transmission modes, host traits - especially their physical condition and immune defenses - may also explain variation in urban susceptibility and responses to pathogens and parasites. The urban environment can be stressful to animals (e.g. food limitation, predators, human activities, heat island effect), resulting in chronic elevation of circulating glucocorticoids (e.g. in white-crowned sparrows (*Zonotrichia leucophrys*) [4] that can impair immunity in birds [64]–[65]. Pollutants (e.g. organochlorines, trace metals) may also negatively affect immune responses of urban animals [66] [67] [68] [69] [70] [71]. Interestingly, in a recent study of urban and rural house sparrows (*Passer domesticus*), feather lead concentration was associated with the degree of infection by one species of avian malaria (*Plasmodium relictum*) [14]. Further studies of urban processes in relation to immune responsiveness and disease susceptibility will shed light on the specific anthropogenic factors that drive susceptibility and responses of urban hosts to parasites and pathogens [5].

One of the factors that can strongly affects immuno-competence in free-living organisms is the levels of oxidative stress. There is now a large body of correlational and experimental evidence in vertebrates showing an increase in the production of reactive oxygen species associated with pathogen-induced inflammation [72]. In addition, activation of the inflammatory response can deplete antioxidants and expose the host to increased risk of oxidative stress [73] [74]. In our study, the levels of oxidative damage in plasma were not associated with the degree of urbanization or viral/coccidial infections. However, consistent with our results, Isaksson *et al.* [53] failed to find significant differences in lipid peroxidation (measured as TBARS) or in the levels of antioxidant enzymes activity in lung between urban and rural great tits (*Parus major*) in Sweden. Isaksson *et al.* [37] did find though that urban great tits had a higher plasma non-enzymatic total antioxidant activity compared to rural birds. Altogether, these results suggest that urban animals can use antioxidants to protect their tissues from urban-derived oxidative stress. These results also point out the need to use several measure of antioxidant capacity or oxidative stress/damage in order to comprehensively understand urban impacts on avian oxidative physiology.

Interestingly, body mass was positively associated with the proportion of land use for agriculture (cultivated vegetation, compacted soil) in our study. Agricultural landscapes are known to be an important source of food (e.g. seed, fruit) for a variety of bird species [75], including house finches [76] [77]. This food may not only be more concentrated than in natural areas but also of higher nutritional quality than backyard bird seeds typical of urbanized areas. Future studies should examine how the different sources of food encountered along the gradient of urbanization may impact house finch physiology and disease resistance.
